# Crosstalk between lung and extrapulmonary organs in sepsis-related acute lung injury/acute respiratory distress syndrome

**DOI:** 10.1186/s13613-025-01513-4

**Published:** 2025-07-14

**Authors:** Bingyu Li, Weishan Lin, Ruomeng Hu, Songjie Bai, Yejiao Ruan, Yushi Fan, Shuya Qiao, Xuehuan Wen, Ruishan Liu, Heyu Chen, Wei Cui, Zhijian Cai, Gensheng Zhang

**Affiliations:** 1 Department of Critical Care Medicine, Second Affiliated Hospital, Zhejiang University School of Medicine, Hangzhou, 310009 China; 2 Spine Center, Xinhua Hospital Affiliated to Shanghai Jiao Tong University School of Medicine, Shanghai, 200092 China; 3 Department of Cardiovascular Surgery, The First Affiliated Hospital, Jiangxi Medical College, Nanchang University, Nanchang, 330006 Jiangxi China; 4 Department of Orthopedics, Institute of Immunology, Second Affiliated Hospital, Zhejiang University School of Medicine, Hangzhou, 310009 Zhejiang China; 5 Department of Oncology, The Affiliated Cangnan Hospital of Wenzhou Medical University, Wenzhou, 325800 Zhejiang China; 6 Key Laboratory of Multiple Organ Failure (Zhejiang University), Ministry of Education, Hangzhou, 310009 China; 7 Department of Critical Care Medicine, Taizhou Hospital of Zhejiang Province affiliated to Wenzhou Medical University, Taizhou, 317000 China

**Keywords:** Sepsis, ALI/ARDS, Organ crosstalk, Extracellular vesicles

## Abstract

Sepsis-related acute lung injury/acute respiratory distress syndrome (ALI/ARDS) is associated with considerable morbidity and mortality, yet the efficacy of current treatments is limited. Previous studies have predominantly focused on the lung itself as an isolated organ, whereas the role of organ crosstalk in the pathogenesis of sepsis-related ALI/ARDS cannot be overlooked. Meanwhile, neglecting the discussion of heterogeneity in sepsis caused by different sources of infection may be another important obstacle to translating previous studies into clinical efficacy. In this review, we initially delineated the distinctions in pathogenesis between pulmonary and extrapulmonary sepsis-related ALI/ARDS in microbial species, pathogenesis, host response, and clinical manifestations. Additionally, systemic organ crosstalk mechanisms are summarized, including the commonality and specificity of systemic inflammation, lung and gut microbiome, as well as cascade cell injury and death in distant organs. Subsequently, organ crosstalk between lung and extrapulmonary in pulmonary sepsis and extrapulmonary sepsis-related ALI/ARDS are discussed by organs, including immunity, neuroendocrine, metabolism, and so forth. Furthermore, extracellular vesicles represent a promising avenue of research as potential players and targets in organ-lung crosstalk in sepsis. While the complexity of multi-organ interactions and the heterogeneity of septic patients present significant challenges, these issues are expected to be addressed by the emergence of organ-on-a-chip platforms, 3D organoid cultures, and multi-omics techniques.

## Introduction

Sepsis-related acute lung injury and acute respiratory distress syndrome (ALI/ARDS) are critical clinical conditions with a high mortality rate, exceeding 30% [1, 2]. These conditions are often accompanied by multi-organ failure, which significantly complicates treatment and prognosis [3].

Crosstalk between the lungs and extrapulmonary organs plays a crucial role in the development of sepsis-related ALI/ARDS. Single organ failure is rare in sepsis; instead, multiple organs are often affected, as evidenced by the widespread use of the Sequential Organ Failure Assessment (SOFA) score for diagnosing sepsis patients [3]. Unfortunately, many previous studies have focused primarily on the changes in the lungs during sepsis-related ALI/ARDS, often neglecting the impact of alterations in other organs on lung injury. Therefore, a deeper understanding of the crosstalk between the lung and extrapulmonary organs is essential for unraveling the complex pathological mechanisms of sepsis-related ALI/ARDS and for developing multi-target, comprehensive treatment strategies.

**Fig. 1:**
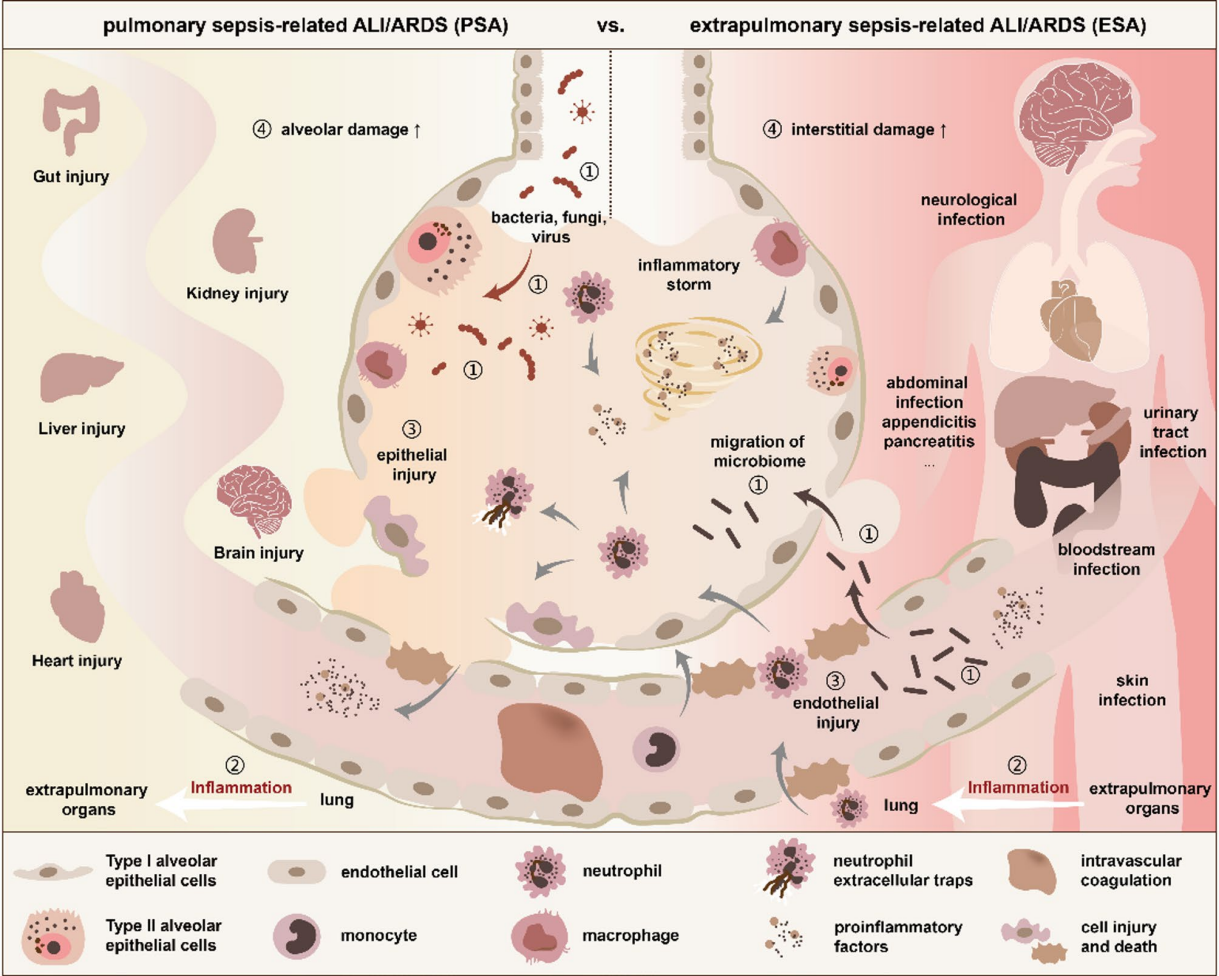
Pulmonary sepsis vs. extrapulmonary sepsis-related ALI/ARDS. Both conditions exhibit the hallmarks of sepsis-related ALI/ARDS, including edema and pneumonia, as well as systemic inflammation and impaired multiorgan function. Regardless of whether it is pulmonary or extrapulmonary, the inflammatory cells primarily involved in the ALI/ARDS condition are neutrophils. However, there are significant differences in microbial species, pathogenesis, host response, and clinical manifestations between PSA and ESA. ① Microbial species: PSA is characterized by an increase in exogenous pathogenic microorganisms and a decrease in the normal respiratory flora in the lungs, whereas ESA results from a shift in the flora, leading to an increase in intestinal microorganisms. ② Pathogenesis: PSA is typically triggered by a lung infection or pneumonia, which subsequently causes damage to other organs. In contrast, ESA is primarily an indirect lung injury resulting from an extrapulmonary infection, mediated by circulating toxic chemicals, autoimmunity, and other factors. ③ Host response: PSA manifests with more severe epithelial injury, while ESA is associated with more pronounced endothelial damage. Compared to ESA, PSA exhibits more intense lung injury and less significant immune suppression. ④ Clinical manifestations: PSA patients are more susceptible to severe alveolar inflammation and pulmonary consolidation, with elevated pulmonary elastic resistance. Conversely, ESA patients are more prone to interstitial lung injury and ground-glass opacities in the lungs, accompanied by higher chest wall elastic resistance

Despite significant investments in both basic and clinical research on the lungs, the most vulnerable target organ in sepsis, since the inception of the Surviving Sepsis Campaign, the prognosis for sepsis-related ALI/ARDS remains unsatisfactory [2, 4]. The heterogeneity arising from different sources of infection may be a key factor contributing to poor therapeutic outcomes, and this issue is garnering increasing attention. Thus, sepsis-related ALI/ARDS is categorized into pulmonary sepsis-related ALI/ARDS (PSA) and extrapulmonary sepsis-related ALI/ARDS (ESA), each characterized by distinct microbial species, pathogenesis, host responses, and clinical manifestations, which could lead to mechanistic differences in organ crosstalk. (Fig. 1) [5–8].

## Systemic organ crosstalk mechanisms between lung and extrapulmonary organs in sepsis-related ALI/ARDS

Before examining the specific organ crosstalk in PSA and ESA, it is essential to discuss the systemic mechanisms involved. These include the commonalities and specificities of systemic inflammation, the lung and gut microbiomes, and the cascade of cell injury and death in distant organs.

### Commonality and specificity of systemic inflammation

#### Commonality: inflammation-barrier dysfunction-hypoxemia axis

Infection in one organ can cause remote organ injury through systemic inflammation, demonstrating critical organ crosstalk. Cytokines (e.g., TNF-α, IL-6) and other soluble mediators extends beyond the primary site via the circulation, triggering systemic inflammation [3, 9]. These toxins activate vascular endothelium, upregulating adhesion molecules (E-selectin, ICAM-1, VCAM-1) to promote leukocyte rolling, adhesion, and transmigration into distal tissues [10]. Cytokines also activate coagulation, leading to a prothrombotic state with widespread microthrombosis [10]. Crucially, resultant microvascular occlusion causes tissue hypoperfusion and ischemic hypoxia, ultimately inducing parenchymal cell death [10].

#### The specificity in the common mechanism

Beyond direct infection spread and inflammation, unique mechanisms contribute to the systemic response, highlighting pathway specificity of the common mechanism. Pulmonary edema impairs lung ventilation, exacerbating hypoxia in other organs. Excretory organs (e.g., liver, kidneys) reduce toxin clearance, while the spleen releases stored cytokines, amplifying inflammation [11, 12]. The brain induces systemic inflammation via the neuroendocrine axis. Cardiac dysfunction impairs perfusion and oxygen delivery, compounding hypoxia and edema.

#### Whether systemic inflammation have organ bias?

An intriguing question is whether systemic inflammation exhibits organ bias, which means certain organs are more prone to accumulating pro-inflammatory substances and are thus more susceptible. While circulating inflammatory factors and damage-associated molecular patterns (DAMPs) diffuse nonspecifically, immune cells and extracellular vesicles (EVs) can target specific sites through chemokine and ligand binding [13, 14]. Additionally, even bacteria aggregation is non-random, but is influenced by tissue physical properties [15]. These mechanisms establish localized pro-inflammatory concentration gradients. It is particularly relevant to consider why the lungs are often the most vulnerable organ in sepsis. This susceptibility may involve unknown mechanisms, warranting further investigation.

### Lung and gut microbiome

Growing evidence indicates the human microbiome orchestrates systemic inflammation and organ dysfunction, regardless of infection origin, forming a self-perpetuating cycle as central mediators.

#### Barrier disruption and vicious cycles

Systemic inflammation and endothelial dysfunction disrupt physiological barriers, facilitating microbial migration between organs [10]. In ESA, gut-vascular barrier leakage and dysbiosis enable gut pathogens to seed the lungs via circulation, while pulmonary endothelial damage in ALI/ARDS permits toxin infiltration into alveoli, exacerbating inflammation [6, 16].

#### Microbial translocation

Dysbiosis of the small intestinal and respiratory microbiomes occurs early in systemic inflammation [17]. Gut-derived pathogens are detected in the alveolar lavage fluid of ALI/ARDS patients but are absent in healthy individuals, suggesting that the gut and lungs interact through microbial translocation [6]. Sequencing confirms presence of normal microbiota in healthy lungs, which is low in biomass but active in functions [18, 19]. The lung microbiota can also disseminate to distant organs and even penetrate the blood-brain barrier (BBB), contributing to neurological disorders [20].

#### Microbial metabolites

Beyond direct microbial migration, microbiome metabolites mediate organ crosstalk. For instance, lung microbiota regulates central nervous system autoimmunity through lipopolysaccharide (LPS) [21, 22]. In T cell-mediated multiple sclerosis, neomycin-induced lung enrichment of LPS-producing phyla primes brain-resident microglia toward a type I interferon-dominant response, thereby suppressing pro-inflammatory cytokine release and reducing T-cell infiltration [22]. Direct pulmonary or cerebral LPS administration recapitulates this protective effect, while neutralizing LPS exacerbates disease [22].

Intestinal flora metabolites also mediate organ crosstalk. Sodium butyrate, for instance, improves survival and protects against sepsis-related ALI/ARDS. Its effect is attributed to increasing regulatory T cells (Tregs), which produce IL-10 and TGF-β, thereby reducing inflammation and enhancing lung and intestine immune barriers [23]. This demonstrates that bacterial metabolites act independently of microbial migration.

#### Convergent mechanisms of immune dysregulation

The microbiota orchestrates organ crosstalk by remodeling the immune microenvironment. Firstly, the microbiota regulates innate immunity. Gut dysbiosis promotes hyper-inflammatory and exhausted T cells production, impairing *Streptococcus pneumoniae* clearance [24]. Crucially, pulmonary microbiota translocation to the brain activates microglia, the resident immune cells of the central nervous system [20]. Secondly, the microbiota shapes adaptive immunity. Gut dysbiosis weakens vaccine responses through secondary bile acids, which promote Th1 cell differentiation but suppress follicular helper T cells, reducing antibody production [25]. Besides, gut microbiota bridges innate and adaptive immunity. For example, *Lactobacillus murinus* induces IL-10 production in macrophages, potentially driving Treg differentiation [26].

#### Therapeutic implications

Targeting microbiota-mediated organ crosstalk presents promising strategies, including barrier protection, selective digestive decontamination, and microbiota restoration. First, barrier protection agents, such as those that enhance gut vascular or pulmonary endothelial integrity, help mitigate microbial translocation [27]. Second, selective digestive decontamination reduces the risk of secondary infections and benefits critically ill patients [28]. Additionally, fecal microbiota transplantation and microbial protection can restore microbial balance. Normal intestinal microbiota can protect against pneumonia by enhancing the function of primary alveolar macrophages [29–32].

### Cascade cell injury and death in distant organ

Not surprisingly, neighboring primary cell death activates bystander death via DAMPs, reactive oxygen species, EVs and proteases [33, 34]. However, whether these mechanisms mediate distant organ crosstalk requires elucidation.

#### Resident immune cell sensing of distant injury

Cell damage and death within one organ can alter the immune environment of distant organs, potentially linking lungs to other organs. Resident macrophages are crucial in this crosstalk by sensing distant organ damages [35, 36]. Following myocardial infarction, stroke, or sepsis, macrophage phenotypic changes (including increased numbers) occur in the heart, lungs, liver, and kidneys [35, 36]. Critically, similar gene modifications occur in macrophages of a given organ after injury to different organs [35, 36], indicating macrophage response is influenced more by their resident organ than by the injury type. These suggest macrophage-mediated organ crosstalk may be systemic. However, whether other cell types exhibit similar functions remains unclear.

#### Domino-like cascade of cell death

Substantial cell interactions can lead to death. Our previous study demonstrated that alveolar macrophages necroptosis during pneumonia can induce necroptosis in unaffected alveolar macrophages [33].

During sepsis, pyroptotic macrophages transmit pyroptosis to neutrophils through mitochondria transferred by microvesicles (a type of EVs), promoting neutrophil death and neutrophil extracellular traps (NETs) formation [37]. Pyroptosis also propagates through EVs transfer of gasdermin pores in immune and non-immune cells [34]. This domino-like cascade of cell death may be a systemic mechanism applicable to most cells in the body. EVs are of particular interest for mediating long-distance cascades, even including BBB crossing [38].

#### Enduring and widespread impact

Distant organ impacts may be long-lasting. Post-sepsis macrophage phenotypic alterations persist for months across multiple organs [35, 36]. Single-cell sequencing shows pro-inflammatory changes in monocytes and macrophages across organs up to three months post-brain damage [39]. However, whether this crosstalk significantly influences distant cells long-term requires investigation.

#### Which mode of injury or death is the key?

In sepsis-related ALI/ARDS, parenchymal and immune cells in lungs and extrapulmonary organs undergo diverse damage and death, including mitochondrial alterations, oxidative stress, autophagy, necrosis, apoptosis, necroptosis, NETosis, and pyroptosis [40, 41]. These common modes suggest a key regulatory target linking lungs and extrapulmonary organs. Identifying the predominant cell death mode in sepsis-related ALI/ARDS and its propagation to distant organs is needed.

## Organ crosstalk between lung and extrapulmonary in PSA

Pneumonia, primarily caused by bacteria, fungi, and viruse infections, is the leading cause of sepsis [5, 41]. Impaired extrapulmonary organs following pneumonia-induced sepsis is common yet often overlooked [42]. The pandemic demonstrated that SARS-CoV-2 infection causes multiple organ failure with long-term sequelae [43–45]. Our multicenter study further showed that respiratory dysfunction progression predicts extrapulmonary organ dysfunction trajectory [46].

### Lung-Brain crosstalk

Beyond cognitive decline upon hospital discharge, ARDS survivors may develop significant long-term brain morbidities, including neurocognitive deficits, lesions, and atrophy [47]. Neurocognitive impairment affects 73% of survivors (46% at 1 year, 47% at 2 years) [47]. Anxiety and moderate-severe depression are prevalent [47].

#### Systemic inflammation-barrier disruption-microbial translocation

Systemic inflammation weakens BBB, allowing toxins to enter the brain [20, 48]. Endogenous bacterial translocation from lungs to brain correlates with post-pneumonia neurological complications, evidenced by shared microbial species [20]. Microglia and astrocytes activation during infections further exacerbates inflammation [20]. Therapeutic strategies include targeting inflammation, repairing the barrier, and remodeling microbiota.

#### Direct neural pathways between the lungs and brains

Direct lung-brain neural pathways exist in pneumonia. TRPV1 + pulmonary sensory neurons link infections to sickness behaviors (such as lethargy and hypothermia) by activating hypothalamic corticotropin-releasing hormone (CRH) neurons in the brain, independent of inflammation [49]. Sickness behaviors reduce disease spread by conserving host energy, signaling the need for treatment, and promoting social distancing and avoidance. This underscores the lung-brain axis in controlling respiratory infection epidemiology.

Microbes can evade detection by encapsulating LPS within exopolysaccharides, causing asymptomatic inflammation. TRPV1 + neuron function depends on LPS detection by toll-like receptor 4 [49]. This suggests therapeutic potential: modulating CRH responses could alleviate acute symptoms, or disrupting biofilms could combat chronic immune evasion.

### Lung-kidney crosstalk

The prevalence of acute kidney injury (AKI) is as high as 27.4% in patients without ARDS and rises to 44.3% in those with ARDS [50].

#### Platelet-dependent injury of kidneys from lungs

Preclinical evidence suggests pneumonia can cause AKI. *P. aeruginosa* pneumonia induced AKI within 24 h, indicated by elevated plasma creatinine and cystatin C [51]. Increaed neutrophil gelatinase-associated lipid transport protein and stable pulse distension suggested direct tubular injury, rather than pre-renal factors [51]. Further experiments demonstrated this lung-kidney crosstalk is platelet-dependent [51]. Platelet-depleted mice exhibited better renal function preservation, and histopathologic analysis ruled out direct bacterial invasion of the kidney and neutrophil infiltration as causes [51]. However, the precise mechanisms of platelet-mediated injury require further investigation. Future research should target platelets to disrupt this connection and mitigate injury.

#### Mechanical ventilation involved damage

While essential for ALI/ARDS management, mechanical ventilation (MV) triples AKI risk [52]. MV’s positive pressure elevates intrathoracic pressure, reducing renal blood flow [53]. Positive end-expiratory pressure may also decrease urine output by increasing renin, aldosterone, and atrial natriuretic peptide [53]. MV-induced alveolar distension triggers adhesion molecule synthesis in lungs and kidneys, promoting renal neutrophil migration and pro-inflammatory cytokine expression [54]. Balancing the optimization of lung protection with the minimization of kidney damage may be a focus of future research.

### Lung-gut crosstalk

#### Pneumonia triggers intestinal injury

Pneumonia triggers intestinal injury through systemic inflammatory cascades. Pathogens and cytokines drive intestinal epithelial apoptosis, mucosal barrier breakdown, and impaired proliferation due to cell cycle arrest [55]. Murine models demonstrate surfactant proteins SP-A/D protect against pneumonia-induced gut injury, while their deletion exacerbates damage [56]. Interventions such as Bcl-2 overexpression or epidermal growth factor ameliorate apoptosis, restore villus architecture, and improve survival [55, 57, 58].

#### Intestinal injury due to pneumonia in turn aggravates lung injury

Pneumonia-induced intestinal injury exacerbates PSA, creating a feedforward loop. Pulmonary inflammation disrupts gut barriers, promoting pathogenic bacterial translocation [6]. Reduced Treg cells and gut short-chain fatty acids also impair alveolar macrophage function, compromising pathogen clearance and amplifying lung injury [23].

#### Therapeutic perspective

Therapeutically, restoring microbiota homeostasis and blocking intestinal apoptosis may disrupt the lung-gut axis. Traditional Chinese medicine recognizes the lung-gut crosstalk, though further mechanistic validation is needed.

### Lung-liver crosstalk

#### Hepatic dysfunction in pneumonia and mechanisms

Hepatic dysfunction affects more than 50% of patients following COVID-19, approximately 60% of those with SARS, and over 60% of individuals with MERS [44, 59, 60]. Multiple mechanisms are involved in the viral pneumonia-induced liver injuries. Firstly, direct virus invasion of hepatocytes or cholangiocytes is evidenced by viral RNA in liver tissues [61–63]. Secondly, sepsis-driven immune dysregulation and systemic damage exacerbate Kupffer cell activation and hepatocyte cycle arrest [63]. Furthermore, agents-induced hepatotoxicity amplifies damage. Protecting liver function during pneumonia and developing hepatically safer antimicrobials are essential.

#### How the liver works in turn on lungs in PSA

In PSA, the liver also impacts the lungs. During infection, the acute phase response, mediated by hepatic NF-κB/STAT3 signaling, facilitates hepatopulmonary crosstalk by reprogramming the blood proteome, with over a thousand transcripts identified as differentially expressed in the liver [64]. Liver-derived acute-phase proteins, including CRP, SAA, and complement components, enhances pulmonary immunity by promoting macrophage phagocytosis and limiting bacterial dissemination [65]. However, dysregulated acute-phase reactions exacerbate systemic inflammation through excessive cytokines and metabolic disturbances [65]. Targeting specific acute-phase pathways or transcriptional regulators such as STAT3 may refine sepsis-related ALI/ARDS therapies.

### Lung-heart crosstalk

In cases of PSA, severe pneumonia can cause acute cardiac injury and remodeling [66]. Beyond systemic inflammation, direct pathogen migrations occurs, forming microlesions that impair cardiac function [67]. Additionally, infiltrated macrophages undergo necroptosis [68]. Cardiomyocyte necrosis in fatal cases suggests that myocardial injury worsens pneumonia [69]. In the long term, the potential for cardiac scarring remains a concern [67–69]. Routine cardiac assessment may be advisable for symptomatic patients, especially elderly or comorbid individuals, warranting further validation.

**Fig. 2:**
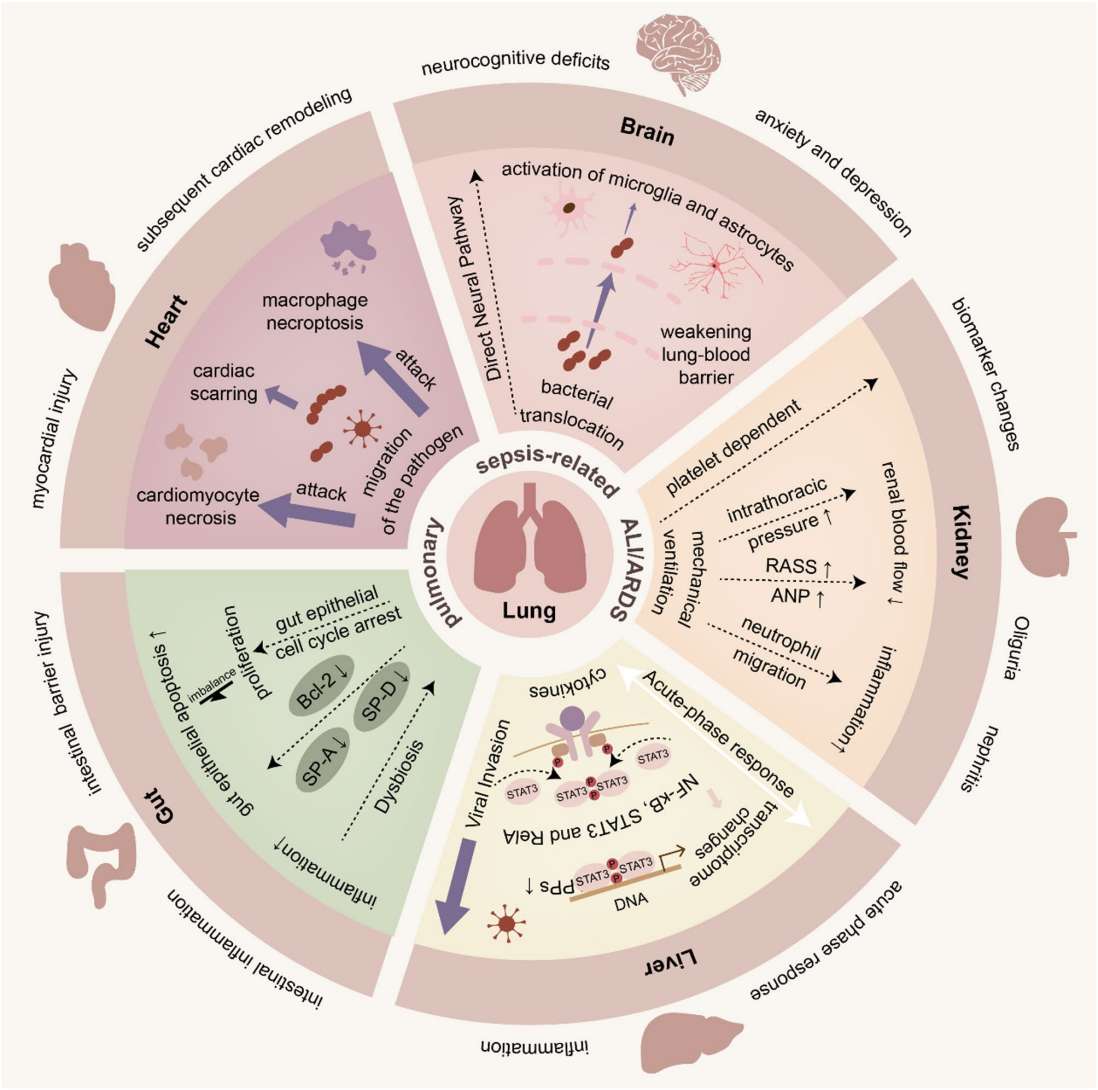
The impact and organ-specific mechanisms of lungs on extrapulmonary organs in pulmonary sepsis-related ALI/ARDS. These crosstalk mechanisms between lungs and other organs including the heart, brain, kidney, liver, and gut are displayed, which mainly include migration and attack of the pathogen, weakening BBB, the influence of mechanical ventilation, the acute phase response and APPs, cell cycle, and so on

In summary, in PSA, lungs damage the heart, brain, kidneys, liver, and gut, through various organ-specific mechanisms, causing extrapulmonary manifestations, as summarized in FIG. 2.

## Organ crosstalk between lung and extrapulmonary in ESA

**Fig. 3:**
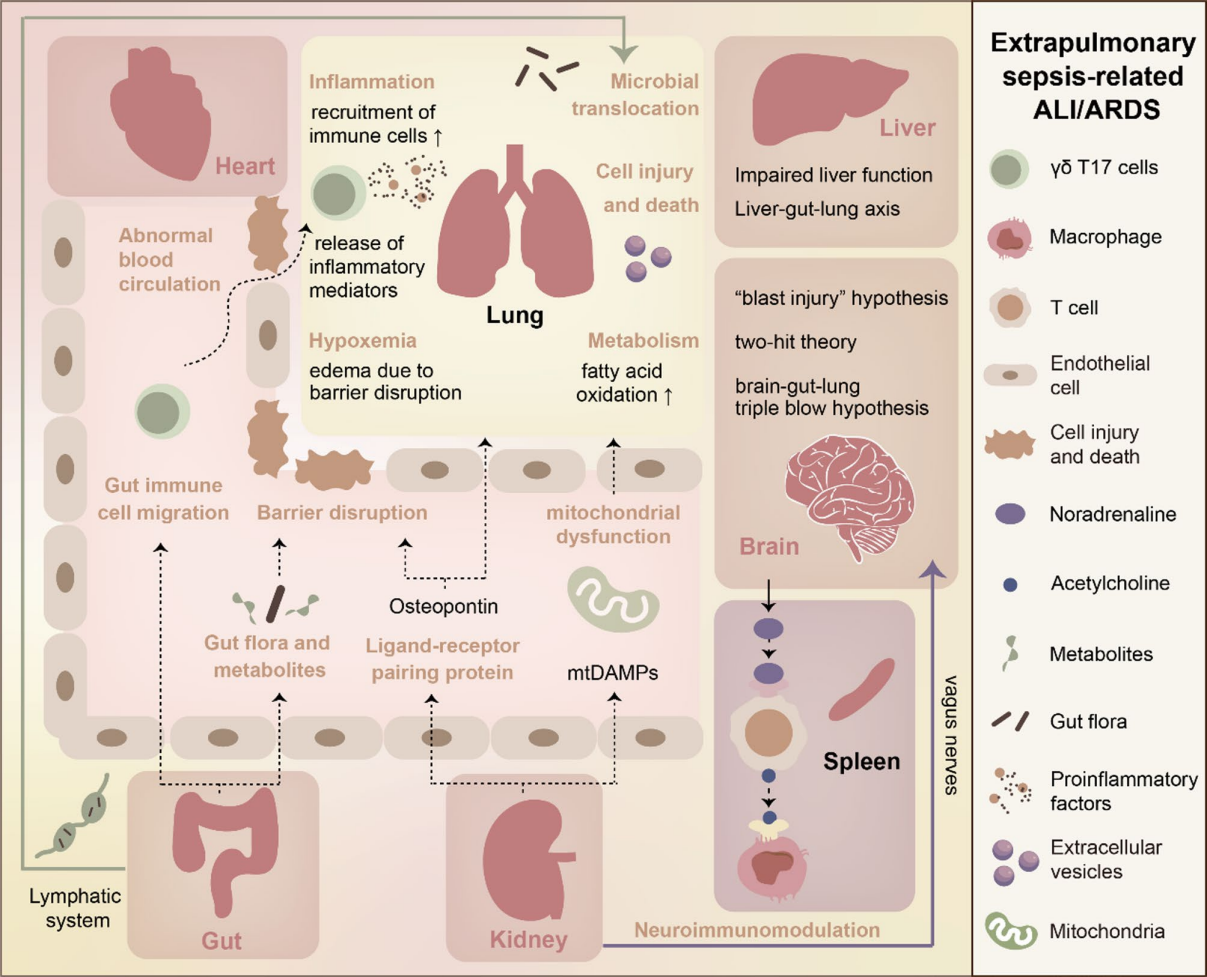
Crosstalk between extrapulmonary organs and lungs in extrapulmonary sepsis-related ALI/ARDS. Inflammation and immunity, metabolism, neural regulation, microbial migration, and other mechanisms are involved. Blood circulation, the lymphatic system, the nervous system, and interstitial spaces may be the transmission pathways of interacting mediators. Extrapulmonary organs not only directly interact with the lungs but also indirectly interact with the lungs through other organs

When sepsis originates outside lungs, it is inappropriate to overlook damage of other organs and their impact on the lungs. The cecal ligation and puncture (CLP) model, widely used and clinically relevant, effectively simulates extrapulmonary sepsis as well as reliably induces lung damage [70]. We focus here on other organs’ effects on the lungs, with Fig. 3 summarizing these crosstalk for clarity.

### Brain-lung crosstalk

Encephalopathy occurs up to 68% of ICU septic patients [71]. Brain injury induces systemic dysfunction, particularly pulmonary vulnerability. Ventilator-associated pneumonia occurs in up to 36% of traumatic brain injury patients, and post-stroke pneumonia remains a leading cause of death in ischemic stroke, emphasizing the critical brain-lung axis [72–74].

**Fig. 4:**
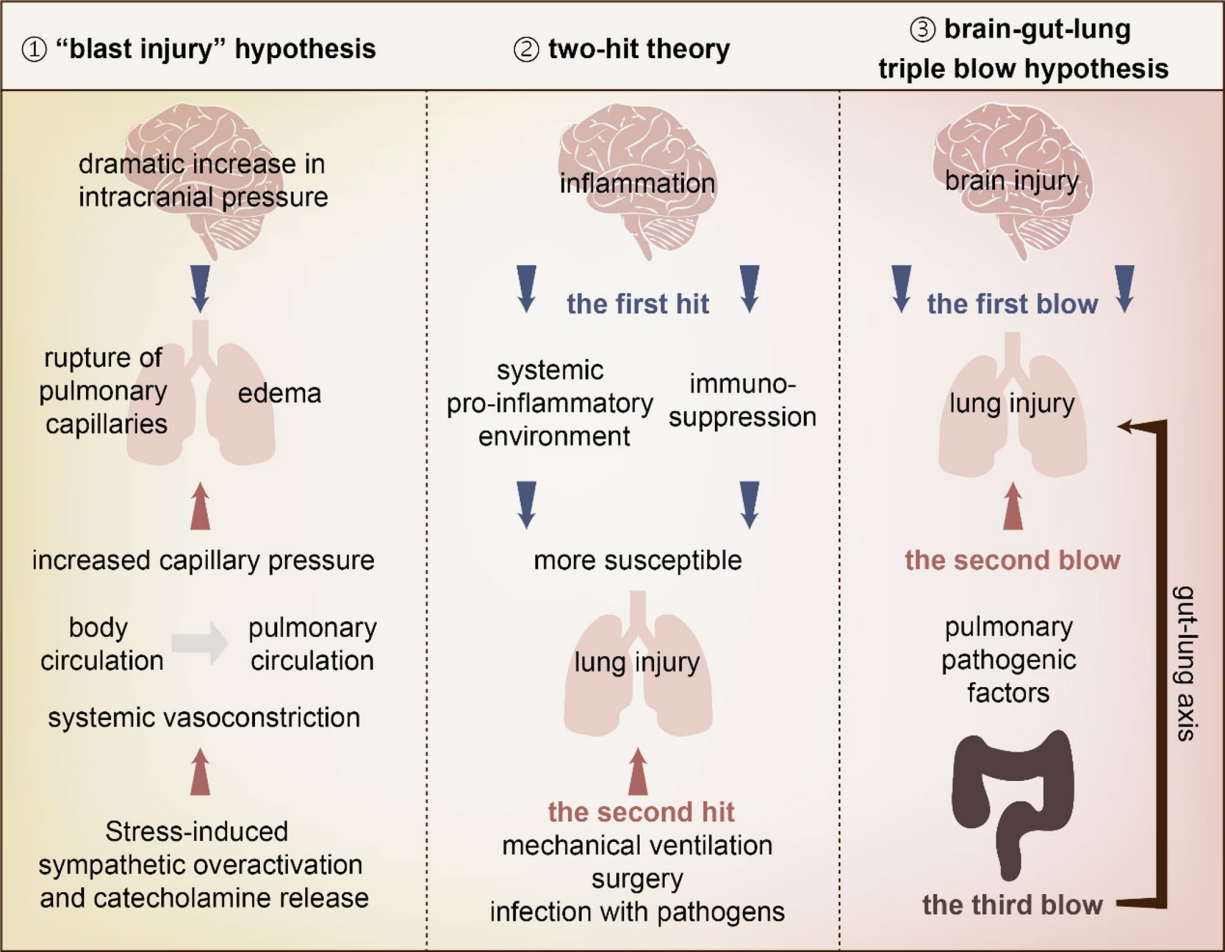
Three hypotheses of brain-lung crosstalk in ESA. (**a**) “Blast injury” hypothesis: Sudden intracranial hypertension elevates pulmonary vascular pressure and thus ruptures pulmonary capillaries. Sympathetic overdrive exacerbates this via catecholamine-induced blood redistribution to the lungs. (**b**) Two-hit theory: Brain injury (first hit) induces systemic inflammation through BBB breach, priming lungs for secondary insults (ventilation, infection; second hit). Concurrent neurogenic immunosuppression increases infection susceptibility. (**c**) Brain-gut-lung triple blow hypothesis: Based on brain injury (one blow) and pulmonary pathogenic factors (the second blow), gut dysbiosis and barrier failure (the third blow) amplify lung inflammation via the gut-lung axis

Three hypotheses delineate brain-lung crosstalk, including the “blast injury” hypothesis, two-hit theory, and brain-gut-lung triple blow hypothesis, which are explained and summarized in FIG 4. The “Blast injury” hypothesis focuses on the hemodynamic model and acute pressure-mediated vascular damage [75]. The two-hit theory emphasizes inflammatory priming and temporal susceptibility [76]. The triple blow hypothesis incorporates the gut microbiome as an injury amplifier [77]. All implicate neurohumoral activation (catecholamines, cytokines) and barrier dysfunction. The hemodynamic hypothesis explains acute edema onset, while inflammatory and microbiome theories account for delayed progression.

Given the established impact of the brain-lung axis, maintaining stable blood pressure, preventing pulmonary infections, and preserving gut microbiota could be beneficial and necessary, warranting further clinical validation.

### Kidney-lung crosstalk

While ALI/ARDS prevalence is often highlighted, it should not be overlooked that the kidney is also a vulnerable organ [78]. Clinical evidence links kidney injury to lung injury and elevated mortality [50, 79–81]. Consensus exists on a lung-kidney communication network, extending beyond systemic inflammation to unique mechanisms.

#### Ligand-receptor pairing protein

Ligand-receptor interactions critically mediate multiorgan failure. Integrated single-cell RNA sequencing and cross-organ ligand-receptor pairing (CellPhoneDB) identified osteopontin (OPN) as a pivotal mediator in AKI-induced ALI [82]. AKI triggers rapid OPN upregulation in tubules and circulation, which correlates with renal dysfunction in mice and patients [82]. In lungs, OPN binds CD44 receptors on endothelial cells and macrophages, disrupting endothelial integrity, triggering vascular leakage and hypoxemia, as well as amplifying inflammation via neutrophil and macrophage recruitment [82].

OPN deletion or neutralization prevented lung injury without mitigating renal pathology [82]. Transplantation of OPN-knockout or injured kidneys into wild-type and healthy mice failed to induce ALI, confirming kidney-specific OPN as the effector [82]. Clinically, elevated serum OPN in AKI patients parallels disease severity, highlighting clinical relevance and therapeutic potential.

#### Metabolism

Metabolic reprogramming occurs early in sepsis, with a shift from oxidative phosphorylation to aerobic glycolysis in renal tubular epithelial cells [83]. Post-AKI, lung metabolomic analysis revealed increased fatty acid oxidation but decreased glycolysis [84]. These changes attributed to mitochondrial dysfunction and mitochondrial DAMPs (mtDAMPs) [84]. Furthermore, circulating mitochondrial dysfunction itself can cause lung injury. Targeting mitochondrial dysfunction offers therapeutic potential for kidney and lung injury in sepsis.

#### Neuro-immunomodulation

Renal afferent vagus nerves activate brain C1 neurons, initiating a neuroimmune anti-inflammatory reflex [85]. This prompts splenic sympathetic noradrenaline release, acting on β2-adrenergic receptors on CD4 + T cells expressing choline acetyltransferase [86]. In response, these T cells release acetylcholine, which activates α7-nicotinic acetylcholine receptors on splenic macrophages to reduce pro-inflammation cytokines and induce anti-inflammatory IL-10 production [86]. Splenic IL-10 could attenuate AKI-induced lung inflammation [86, 87].

Enhancing the acetylcholine-mediated anti-inflammatory IL-10 production in the spleen may serve as a pivotal translational target. Clinical trials using the cholinergic anti-inflammatory pathway show favorable outcomes for lung injury [88]. However, inappropriate immunosuppression may be detrimental. A case in point is that during the immunosuppressive phase of sepsis, depletion of splenic neutrophils (major IL-10 source) may paradoxically improve survival [89].

### Gut-lung crosstalk

Despite the established gut microbial translocation and metabolite-mediated gut-lung axis through circulation and lymph system in sepsis-associated ALI/ARDS, the direct migration of gut immune cells to the lungs remains a surprising phenomenon. In an ESA model induced by cecal ligation and puncture, alveolar macrophages that have activated Wnt signaling upregulate CCL1, which promotes γδ T17 cells to migrate from the small intestine to the lung, where they create an inflammatory response in mice that is dominated by IL-17 A [90]. According to the study, CD44 + Ly6C- IL-7Rhigh CD8low cells are the main migratory subtype exacerbating ALI [90]. Further investigation is required to elucidate the role of other immune cell types in extrapulmonary sepsis, particularly in the context of gut-origin sepsis.

### Liver-lung crosstalk

#### Impaired liver functions: detoxification, immunity, coagulation and metabolism

Impaired liver functions, including detoxification, immunity, coagulation, and metabolism contribute to pulmonary injury. Firstly, impaired hepatic detoxification leads to the systemic accumulation of toxins, while reduced Kupffer cells phagocytosis impairs pathogen clearance, amplifying systemic inflammation and pulmonary endothelial and alveolar damage [91, 92]. Secondly, diminished coagulation factors synthesis and anticoagulant imbalance exacerbate hypercoagulability, promoting pulmonary microthrombosis and platelet activation, worsening lung injury [93]. Furthermore, detachment of NETs from hepatic vasculature and pulmonary embolization cause vascular occlusion [94]. Additionally, bile acid accumulation suppresses pulmonary surfactant synthesis, inducing alveolar collapse and impaired oxygenation [95].

#### Indirect role of the liver-gut-lung axis

The liver also impacts lungs indirectly through liver-gut-lung axis [96]. The liver communicates with the gut through bile, portal veins, and systemic circulation [96, 97]. Microbial danger signals resulting from gut injury can be sensed by the liver [97]. In turn, hepatic Kupffer cell dysfunction and cholestasis in the liver exacerbate gut barrier failure, potentially further injuring the lungs via gut flora and toxins [98].

### Heart-lung crosstalk

Systemic inflammation, circulatory abnormalities, and metabolic abnormalities are possible mechanisms. Similar to other organ-lung crosstalk, inflammation may mediate cardiopulmonary interactions. Cardiac dysfunction may lead to systemic and microcirculatory circulatory abnormalities, exacerbating pulmonary hypoperfusion and hypoxia [99]. Metabolic dysfunction caused by oxygen deprivation also mediates cardiopulmonary interactions. Mitochondrial dysfunction drives multi-organ impairment in sepsis [100]. ALI/ARDS features intractable hypoxemia, while septic cardiac injury involves mitochondrial respiration failure and causing abnormal myocardial metabolism and contractile dysfunction [101]. Crosstalk between hearts and lungs remain to be elucidated.

In summary, the crosstalk between the lung and extrapulmonary organs in PSA and ESA has been summarized in Table 1.

**Table 1:** Crosstalk between lung and extrapulmonary organs in PSA and ESA

Mechanism Category	Key Mechanism Description	Ref
***A. Systemic Organ Crosstalk Mechanisms***		
Commonality and Specificity Of Systemic Inflammation	**Commonality**: Inflammation-barrier dysfunction-hypoxemia axis drives multi-organ injury (epithelial/endothelial damage, microvascular dysfunction).	[10, 40]
	**Specificity**: Organ-specific amplification of injury - Lungs: Edema exacerbates extrapulmonary hypoxia. - Liver/kidney: Reduced toxin clearance. - Brain: Neuroendocrine axis activation. - Heart: Perfusion deficits worsen hypoxia.	[11, 12]
	**Organ Bias**: Localized inflammatory gradients mediated by immune cells, EVs, and chemokines (lung vulnerability remains unclear).	[13–15]
Lung and Gut Microbiome	**Microbial translocation**: - Gut pathogens detected in alveolar lavage - Lung microbiota detected in brain	[6, 20]
	**Microbial Metabolites**: - Lung microbiota: LPS primes brain microglia for neuroprotection. - Gut microbiota: Sodium butyrate enhances Tregs, reducing inflammation.	[21–23]
	**Immune Dysregulation**: Microbiota remodel innate/adaptive immunity	[24–26]
	**Therapeutic Strategies**: Barrier protection, selective digestive decontamination, fecal microbiota transplantation.	[27, 29–32]
Cascade Cell Injury and Death	**Resident Immune Sensing**: Macrophages in distant organs adopt injury-specific phenotypes (persisting for months post-sepsis).	[35, 36]
	**Domino-like Cell Death**: EVs transfer pyroptotic signals (gasdermin pores, mitochondria) to distant cells.	[33, 34, 37, 38]
	**Which is the Key Modes**: Dominant pathways undefined	
***B. Organ Crosstalk between lung and extrapulmonary in PSA***		
Brain	**Systemic Inflammation-Microbial translocation**: BBB disruption allows microbial/toxin entry (same lung-brain microbiota); and microglial activation.	[20, 48]
	**Direct Neural Pathway**: TRPV1 + lung sensory neurons activate hypothalamic CRH neurons, driving sickness behaviors (independent of cytokines).	[49]
Kidney	**Platelet-Dependent Injury**: Pneumonia-induced AKI mediated by platelets.	[51]
	**MV**: MV increases renal adhesion molecules/pro-inflammatory cytokines and reduces renal blood flow.	[52–54]
Gut	**Pneumonia**→**Gut Injury:** Cytokine storm induces intestinal apoptosis/barrier breakdown; SP-A/D and EGF mitigate damage.	[55–58]
	**Gut**→**Lung Injury:** Dysbiosis reduces Tregs and SCFAs, impairing alveolar macrophage function.	[23]
Liver	**Viral Invasion**: Direct hepatocyte/cholangiocyte infection (SARS-CoV-2, MERS-CoV); Kupffer cell activation.	[61–63]
	**Acute-Phase Response**: Hepatic NF-κB/STAT3 signaling produces acute phase proteins (e.g., CRP), enhancing lung immunity.	[64, 65]
Heart	**Pathogen Translocation**: Streptococcus pneumoniae causes myocardial microlesions; SARS-CoV-2 infects cardiac pericytes.	[67–69]
	**Clinical Impact**: Myocardial necrosis predicts poor prognosis	[69]
***C. Organ Crosstalk between lung and extrapulmonary in ESA***		
Brain	**Injury Hypotheses**: 1. Blast Injury: Intracranial hypertension→pulmonary capillary rupture. 2. Two-Hit Theory: Brain injury primes lungs for secondary insults. 3.Triple Blow: Brain injury + gut dysbiosis→amplified lung inflammation.	[75–77]
Kidney	**Ligand-receptor pairing protein (OPN-CD44) Axis**: AKI upregulates OPN, binding pulmonary CD44 to disrupt endothelial integrity.	^[0]^
	**Neuro-Immunomodulation**: Renal vagal afferents activate splenic anti-inflammatory pathways.	[85–87]
Gut	**Gut microbial translocation and metabolite** Remodel innate/adaptive immunity	[26]
	**Immune Cell Migration**: Gut-derived γδ T17 cells migrate to the lungs via CCL1 signaling, driving IL-17 A-dominated inflammation.	[90]
Liver	**Impaired Hepatic Functions**: Detoxification/immune/coagulation/metabolic dysfunction→toxin accumulation, microthrombosis, bile acid-induced alveolar collapse.	[91–95]
	**Liver-Gut-Lung Axis**: Cholestasis and Kupffer cell dysfunction exacerbate gut barrier failure, amplifying microbial translocation.	[96–98]
Heart	**Circulatory/Metabolic Dysfunction**: Reduced cardiac output→pulmonary hypoxia; mitochondrial dysfunction links cardiopulmonary injury.	[99–101]

## EVs as promising fields for crosstalk between lung and extrapulmonary organs in sepsis-related ALI/ARDS

**Fig. 5:**
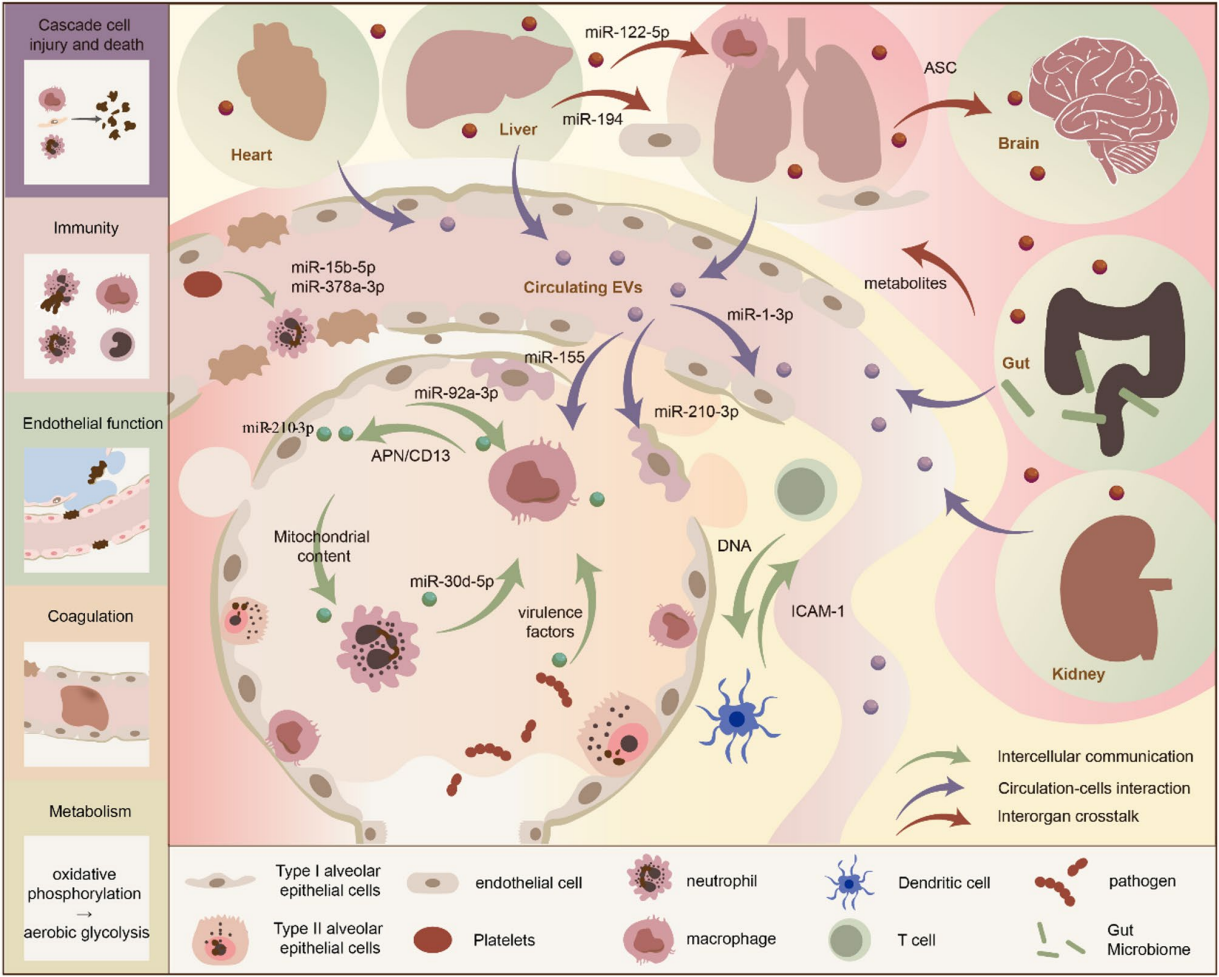
The role of EVs in organ crosstalk in sepsis-related ALI/ARDS. Systemic mechanisms, including a cascade of cell injury and death, immunity, endothelial function, metabolism, and coagulation, as well as communications facilitated by EVs are included

EVs, including exosomes, microvesicles, microsomes, apoptotic bodies, and other subtypes, are membrane-bound structures released by cells, wrapping cargo (including proteins, genetic material, lipids, soluble mediators, etc.) [102]. The role of EVs in organ crosstalk in sepsis-related ALI/ARDS is summarized in FIG 5, including mediating systemic mechanisms (a domino-like cascade of cell injury and death described in Sect. 1, immunity, endothelial function, metabolism, and coagulation) and facilitating both short-range and long-range communication.

Clinical evidence links EVs to sepsis severity. Plasma EVs positively correlate with SOFA score, and higher plasma exosome levels associated with increased 90-day mortality in sepsis [103]. Microvesicles are threefold higher in septic shock patients versus healthy individuals [104].

### Systemic mechanisms meditated by EVs

Beyond mediating cell death cascades (Sect. 1), EVs meditate immunity, endothelial function, metabolism, and coagulation in sepsis. For immunity, EVs deliver LPS, which is crucial for inflammation [105]. EVs can also modulate immune cell functions by directing neutrophil recruitment and migration, influencing macrophage polarization, aiding T and B cell maturation, as well as improving antigen presentation [106–109]. In addition, EVs can directly impair vascular barrier function [110]. For metabolism, the shift from oxidative phosphorylation to aerobic glycolysis is a key mechanism of host defense during the acute phase of sepsis [111]. In septic mice, EVs can act on SOCS1, a metabolic reprogramming regulator, potentially influencing inflammation and organ damage [112, 113]. EVs also promote thrombosis, which is not only related to exposure to tissue factors and phosphatidylserines but also involves complex cross-linking of cytokines [114].

### Short-range and long-range communications facilitated by EVs

Research has elucidated that EVs can mediate short-range intercellular interactions and play a significant role in organ dysfunction caused by sepsis and inflammatory diseases, which are summarized in Table 2. Furthermore, EVs have been found to mediate communication between distant tissues and organs, indicating that possible transport pathways for EV-mediated organ crosstalk exist. Besides, affected circulating EVs exert damaging effects on diseases, suggesting that the bloodstream may serve as a transport pathway for functional EVs. Relevant evidence and references are also summarized in Table 2.

**Table 2:** EVs-mediated short-range and long-range communications in sepsis and inflammatory diseases

Crosstalk	Source Cell/Organ	Target Cells/Organs	Key Components	Mechanism
Intercellular communication (short-range)	*Streptococcus pneumoniae*	Alveolar epithelial cells	virulence factors	Alveolar epithelial barrier disruption [115]
	Alveolar Epithelial Cells	AMs	miR-92a-3p	Alveolar macrophage activation [116]
		Neutrophils	Mitochondrial content	Neutrophil oxidative suppression [117]
	Neutrophils	Macrophages	miR-30d-5p	M1 polarization and pyroptosis [108]
		Extracellular matrix in the lung	integrin Mac-1 and neutrophil elastase	Matrix destruction [118]
		Intestinal epithelial cells	MMP-9	Disruption of epithelial intercellular adhesions [119]
	Macrophages	Hepatic stellate cells	miR-103-3p	Liver fibrosis [120]
		Lung epithelial cells	APN/CD13	Lung epithelial cells necroptosis [121]
		Cardiac fibroblast	miR-155	Suppress fibroblast proliferation and promote inflammation [122]
	Dendritic cells	T cells	ICAM-1	T cell activation and differentiation [123]
	T cells	Dendritic cells	DNA	Antiviral responses [124]
	Platelets	Neutrophils	HMGB1, miR-15b-5p, miR-378a-3p	NETs formation [125]
			12-LOX, sPLA2-IIA	Pro-inflammatory responses [126]
			caspase-1, IL-1β	Lung vasoocclusion [127]
	Tubular Epithelial Cells	Renal macrophages	miR-23a, miR-19b-3p	M1 activation and inflammation [128, 129]
	Hepatocytes	Liver macrophages	miR-192-5p	M1 polarization inflammation [130]
Circulation-cells interaction	Peripheral blood from ALI mice	Macrophages	miR-155	Macrophage proliferation and inflammation [113]
	Serum after LPS challenge	CNS microglia/astrocytes	miR-155	Neuroinflammation [131]
	Sepsis plasma	Endothelial cells	miR-1-3p	Weakens vascular barrier function [110]
	Plasma of septic patients	Macrophages, epithelial cells, endothelial cells	miR-210-3p	Autophagy and inflammation [132]
	Serum of burn injury patients	Lung (PMVECs)	S100A9	Microvascular hyperpermeability [133]
	Plasma after experimental acute pancreatitis	Lung (AMs)	unclear	M1 polarization inflammation [134]
	Serum with acute pancreatitis	Lung (PMVECs)	ITGAM, ITGB2	Inflammation and disruption of the endothelial barrier [135]
Interorgan crosstalk (long-range)	lung	bone marrow	dsDNA	Neutrophil recruitment [136]
	liver	Lung (AMs)	miR-122-5p	M1 polarization inflammation [137]
	Liver (hepatocytes)	Lung (PMVECs)	miR-194	Angiogenesis [138]
	Lung (AMs)	Brain	ASC	Pyroptosis and necroptosis in the hippocampus [139]
	Gut(Lactobacillus paracasei)	Lung (airway epithelium)	metabolites	Inhibition of IL-8 production [140]

AMs: Alveolar Macrophages, PMVECs: pulmonary microvascular endothelial cells, ASC: apoptosis-associated speck-like protein containing a caspase recruitment domain

Given the established role of EVs in intercellular communication, and the emerging evidence for their long-distance communication, it is plausive that EVs are potential mechanisms and promising targets for crosstalk between lung and extrapulmonary organs in sepsis-related ALI/ARDS [141, 142]. Despite the numerous related studies that have been conducted, definitive direct evidence is still unsubstantial. However, this area presents a highly promising direction for future research, warranting further exploration.

## Future directions and research challenges

Future research on organ-lung crosstalk in sepsis should focus on several key areas. First, deeper explorations of signaling pathways, regulatory factors, and epigenetic modifications are needed to understand inter-organ communication. Second, comprehensive studies must elucidate the temporal dynamics of organ dysfunction and crosstalk during sepsis. Third, translational research is essential to develop targeted therapies that can be validated in clinical settings. Finally, large-scale clinical trials are required to evaluate the efficacy and safety of new treatments and identify personalized strategies.

However, significant challenges remain. The animal models poorly replicate human multiorgan complexity. Clinical heterogeneity complicates the identification of biomarkers and therapeutic targets. Ethical considerations also pose obstacles to prospective studies. Despite these challenges, emerging approaches offer hope. New experimental models like organ-on-a-chip and 3D organoids can better simulate organ interactions. Omics techniques and interdisciplinary collaborations offer promising avenues for improved understanding and therapies.

## Conclusions

Crosstalk between lungs and extrapulmonary organs is critical in sepsis-related ALI/ARDS, regardless of the source of infection. While studies were organ-centered due to technological limits in the past, multidisciplinary progress is shifting focus to organ interactions to improve therapeutic efficacy.

## Data Availability

Not applicable.

## Abbreviations

ALI/ARDS: Sepsis-related acute lung injury/acute respiratory distress syndrome
SOFA: Sequential Organ Failure Assessment
PSA: Pulmonary sepsis-related acute lung injury/acute respiratory distress syndrome
ESA: Extrapulmonary sepsis-related acute lung injury/acute respiratory distress syndrome
DAMPs: Damage-associated molecular patterns
EVs: Extracellular vesicles
BBB: Blood-brain barrier
LPS: Lipopolysaccharide
Treg: Regulatory T cell
NETs: Neutrophil extracellular traps
CRH: Corticotropin-releasing hormone
AKI: Acute kidney injury
MV: Mechanical ventilation
OPN: Osteopontin
mtDAMPs: Mitochondrial damage-associated molecular patterns
